# *Demequina capsici* sp. nov., a novel plant growth-promoting actinomycete isolated from the rhizosphere of bell pepper (*Capsicum annuum*)

**DOI:** 10.1038/s41598-024-66202-x

**Published:** 2024-07-09

**Authors:** Zalfa Humaira, Donghyun Cho, Yuxin Peng, Forbes Avila, Yu Lim Park, Cha Young Kim, Jiyoung Lee

**Affiliations:** 1 Korean Collection for Type Cultures (KCTC), Biological Resource Center, Korea Research Institute of Bioscience and Biotechnology (KRIBB), Jeongeup, Jeollabuk-do 56212 Republic of Korea; 2 Department of Biosystems and Bioengineering, KRIBB School of Biotechnology, University of Science and Technology (UST), Yuseong, Daejeon, 34113 Republic of Korea; 3 Animal Model Research Group, Jeonbuk Branch Institute, Korea Institute of Toxicology, Jeongeup, Jeollabuk-do 56212 Republic of Korea; 4 Human and Environmental Toxicology, University of Science and Technology (UST), Yuseong, Daejeon, 34113 Republic of Korea

**Keywords:** *Demequina*, Rhizosphere, PGPB, Auxin, Novel species, Microbiology, Plant sciences

## Abstract

*Demequina*, commonly found in coastal and marine environments, represents a genus of Actinomycetes. In this study, strains *Demequina* PMTSA13^T^ and OYTSA14 were isolated from the rhizosphere of *Capsicum annuum,* leading to the discovery of a novel species, *Demequina capsici*. Bacteria play a significant role in plant growth, yet there have been no reports of the genus *Demequina* acting as plant growth-promoting bacteria (PGPB). Comparative genomics analysis revealed ANI similarity values of 74.05–80.63% for PMTSA13^T^ and 74.02–80.54% for OYTSA14, in comparison to various *Demequina* species. The digital DNA-DNA hybridization (dDDH) values for PMTSA13^T^ ranged from 19 to 39%, and 19.1–38.6% for OYTSA14. Genome annotation revealed the presence of genes associated with carbohydrate metabolism and transport, suggesting a potential role in nutrient cycling and availability for plants. These strains were notably rich in genes related to ‘carbohydrate metabolism and transport (G)’, according to their Cluster of Orthologous Groups (COG) classification. Additionally, both strains were capable of producing auxin (IAA) and exhibited enzymatic activities for cellulose degradation and catalase. Furthermore, PMTSA13^T^ and OYTSA14 significantly induced the growth of *Arabidopsis thaliana* seedlings primarily attributed to their capacity to produce IAA, which plays a crucial role in stimulating plant growth and development. These findings shed light on the potential roles of *Demequina* strains in plant–microbe interactions and agricultural applications. The type strain is *Demequina capsici* PMTSA13^T^ (= KCTC 59028^T^ = GDMCC 1.4451^T^), meanwhile OYTSA14 is identified as different strains of *Demequina capsici*.

## Introduction

The rhizosphere, the underground area surrounding plant roots, is where complex associations and interactions occur between plant roots and other microorganisms^1^. These interactions are facilitated by the production of root exudates by plants, which play a crucial role in chemical communication, predominantly influencing the composition and activity of microorganisms in the rhizosphere^2,3^. On the other hand, microorganisms in the soil interpret the signals they receive and release signaling molecules in response, capable of influencing their plant hosts^4^. The communication pathways between plants and microorganisms in the rhizosphere have primarily been investigated within close symbiotic relationships, which also include mutually beneficial interactions^5^. One of the primary outcomes of these beneficial interactions is plant protection against biotic and abiotic stresses, as well as the promotion of plant growth. This action can be achieved through the presence of beneficial bacteria, or plant growth-promoting bacteria (PGPB), in the rhizosphere.

PGPB belong to a heterogeneous group of bacteria species which can be found as an endophyte, symbiotic, associative, or free living in the plants, especially root and rhizosphere area in this context. PGPBs promote their host’s growth through various mechanisms. Notably, PGPBs-rhizobia, such as *Bradyrhizobium japonicum*^6^, are known for biological nitrogen fixation. Other important mechanisms of PGPBs-rhizobia include the production of indolic compounds such as the auxin phytohormone indole-3-acetic acid (IAA), siderophore production, ACC deaminase activity, and phosphate solubilization^7^.

Actinomycetes, a group of fungi-like gram-positive bacteria, are ubiquitous in various ecosystems, particularly in soil. This phylum is well-known for its ability to produce secondary metabolites such as antibiotics and growth-promoting substances, as well as enzymes, making it important for agriculture by playing a significant role in soil health and plant growth^8^. The actinomycete bacterium from the genus *Demequina* was first identified and isolated from tidal flat sediment in South Korea by Yi et al. ^9^ with *Demequina aestuarii* as the type species. As of the latest update on December 11, 2023, approximately 24 species within the *Demequina* genus have been identified (https://lpsn.dsmz.de/genus/demequina), predominantly inhabiting marine-related ecosystems and exhibiting halotolerant characteristics. One of the main characteristics of this genus is the presence of demethylmenaquinone (DMK) on its plasma membranes^9–11^, which is involved in nitrate respiration^12^*. Demequina* has been reported to possess significant capabilities for degrading complex carbon sources, nitrite ammonification, sulfide oxidation, and potential involvement in polyketide biosynthesis^13^. The typical characteristics of the *Demequina* genus, commonly found in coastal ecosystems, suggest its potential resilience in varying salinity conditions^13^. Despite these characteristics, there is limited literature on the potential role of *Demequina* as PGPB. Consequently, this bacterium may play a role in rhizosphere relationships with crop plants, emphasizing the need for further exploration into its ecological role. The main objective of this study is to explore and characterize the role of *Demequina capsici* sp. nov., a novel actinomycete isolated from the rhizosphere of bell pepper (*Capsicum annuum*), in promoting plant growth within the rhizosphere.

## Results and discussion

### 16S rRNA phylogenetic analysis

**Figure 1:**
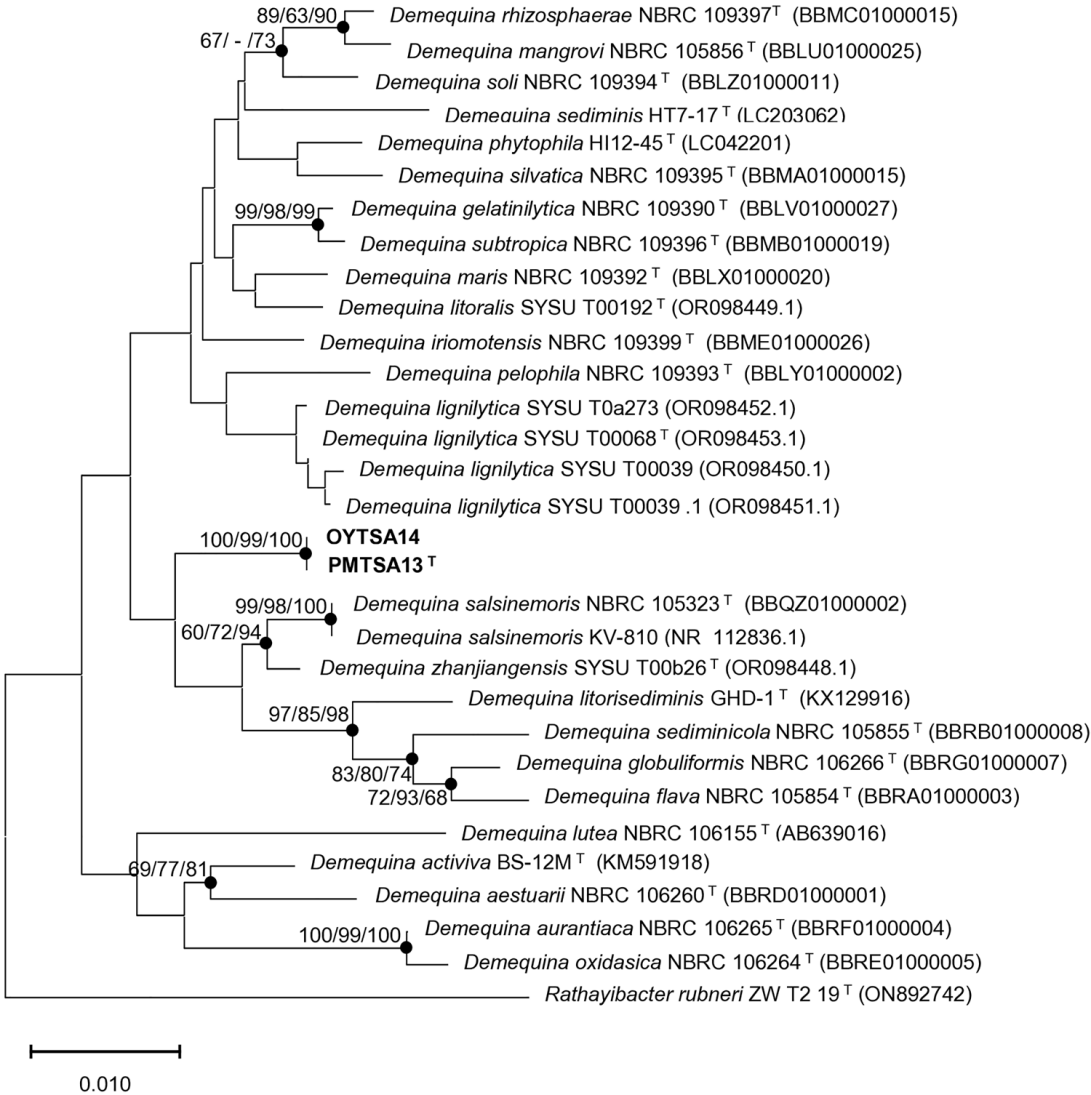
Neighbor-joining (NJ) phylogenetic tree based on 16S rRNA gene sequences of strains PMTSA13^T^ , OYTSA14, and its closely related species of *Demequina*. Bootstrap values (> 60%) were calculated using the NJ, maximum-likelihood (ML), and minimum evolution (ME) algorithms. Filled circles denote nodes that appeared in ML and ME algorithms. *Rathayibacter rubneri* ZW T2 19^T^ was used as an outgroup. The scale bar represents 0.01 substitutions per nucleotide position.

Based on 16S rRNA gene sequences comparative analysis, PMTSA13^T^ and OYTSA14 exhibited the highest sequence similarity with *Demequina iriomotensis* NBRC 109399^T^ (98.06%), followed by *Demequina salsinemoris* NBRC 105323^T^ (97.99%), and *Demequina pelophila* NBRC 109393^T^ (97.64%). Given that the sequence similarity falls below the threshold values of 98.60%^14^, PMTSA13^T^ and OYTSA14 were classified as new species of the genus *Demequina*. To elucidate the phylogenetic relationships, a Neighbor-Joining (NJ) phylogenetic tree was reconstructed as the backbone, supported with Maximum Likelihood (ML) and Maximum Parsimony (MP) methods (Fig. 1). The phylogenetic tree revealed that PMTSA13^T^ and OYTSA14 form a distinct clade, closely clustering with *D. salsinemoris* NBRC 105323^T^ and KV-810, *D. zhanjiangensis* SYSU T00b26^T^, *D. litorisediminis* GHD-1^T^, *D. sediminicola* NBRC 105855^T^, *D. globuliformis* NBRC 106266^T^, and *D. flava* NBRC 105854^T^.

### Genome features and phylogenomic analysis

**Figure 2:**
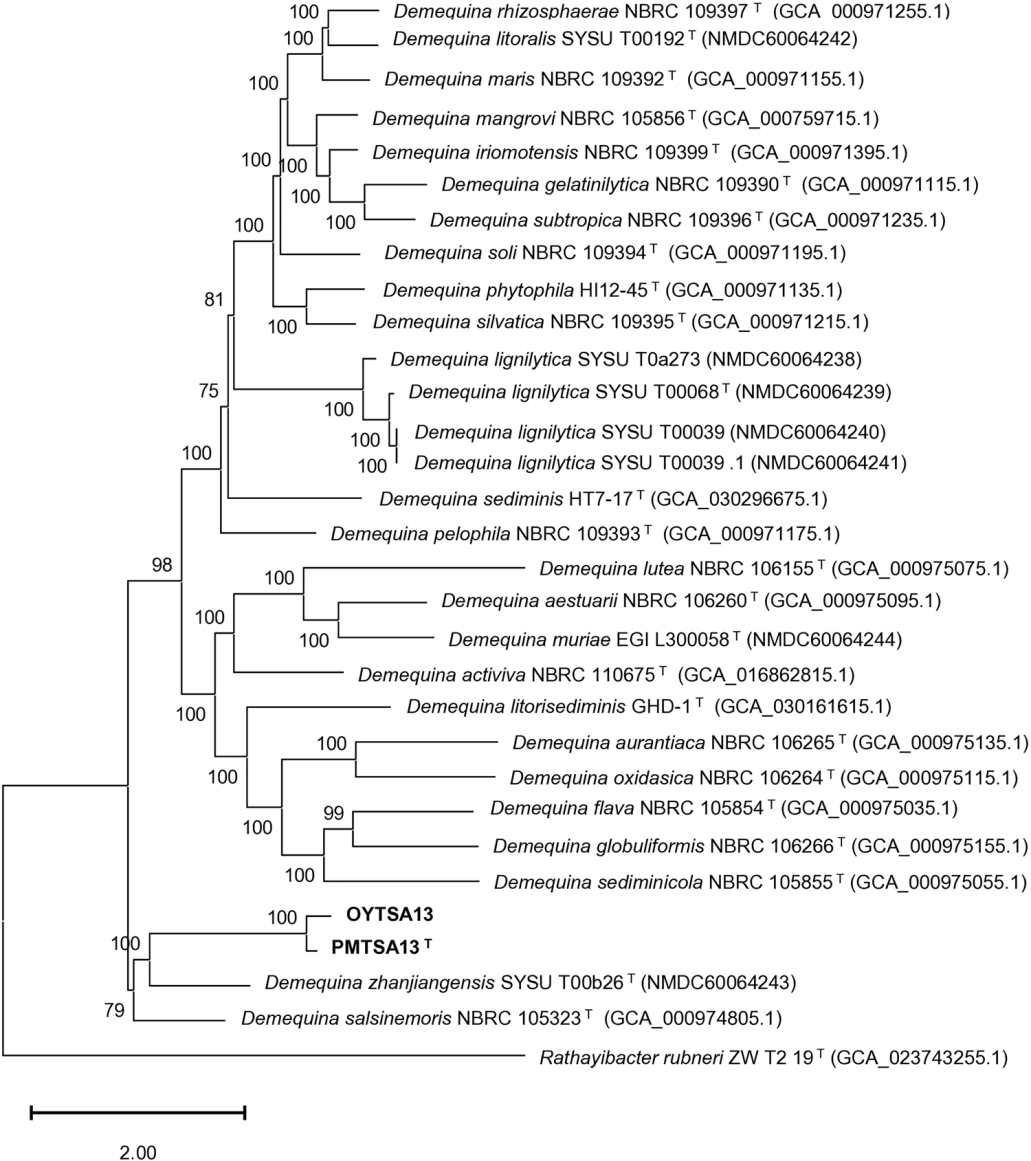
ML tree based on whole genome sequences of PMTSA13^T^ , OYTSA14, and its closely related species of *Demequina*. Bootstrap values (> 60%) were shown on the nodes with 1000 replicates. *Rathayibacter rubneri* ZW T2 19^T^ was used as an outgroup. The scale bar represents 2.00 substitutions per nucleotide position.

Total genome size of PMTSA13^T^ is 3,083,245 bp with a GC content of 69.8%, while OYTSA14 is 3,085,583 bp and GC content of 69.7% (Fig. S1). Genome annotation revealed that the PMTSA13^T^ and OYTSA14 genomes contain 2881 and 2868 coding sequence (CDS), 48 and 50 tRNA genes, and 6 rRNA genes, respectively. ANI similarity values for PMTSA13^T^ compared to *D. zhanjiangensis* SYSU T00b26^T^, *D. salsinemoris* NBRC 105,323 T, *D. litorisediminis* GHD-1^T^ and *D. aestuarii* NBRC 106,260^T^ ranged from 74.05% to 80.63%, and from 74.02% to 80.54% for OYTSA14. Meanwhile, the dDDH value for PMTSA13^T^ is 19–39% and 19.1–38.6% for OYTSA14, which is lower than ANI (~ 95%) and dDDH (70%) classification threshold^15^. This indicates the distinctiveness of PMTSA13^T^ and OYTSA14 from other species of *Demequina*, suggesting that they belong to different species. Furthermore, PMTSA13^T^ and OYTSA14 were different strain with OrthoANI values 98.81% and dDDH 0.01% (Fig. S2). Phylogenomic tree constructed with UBCG (version 3.0) revealed distinct lineage between PMTSA13^T^ and OYTSA14 against *D. zhanjiangensis* SYSU T00b26 ^T^ and *D. salsinemoris* NBRC 105323^T^ (Fig. 2), consistent with 16s sequence phylogenetic tree.

**Figure 3:**
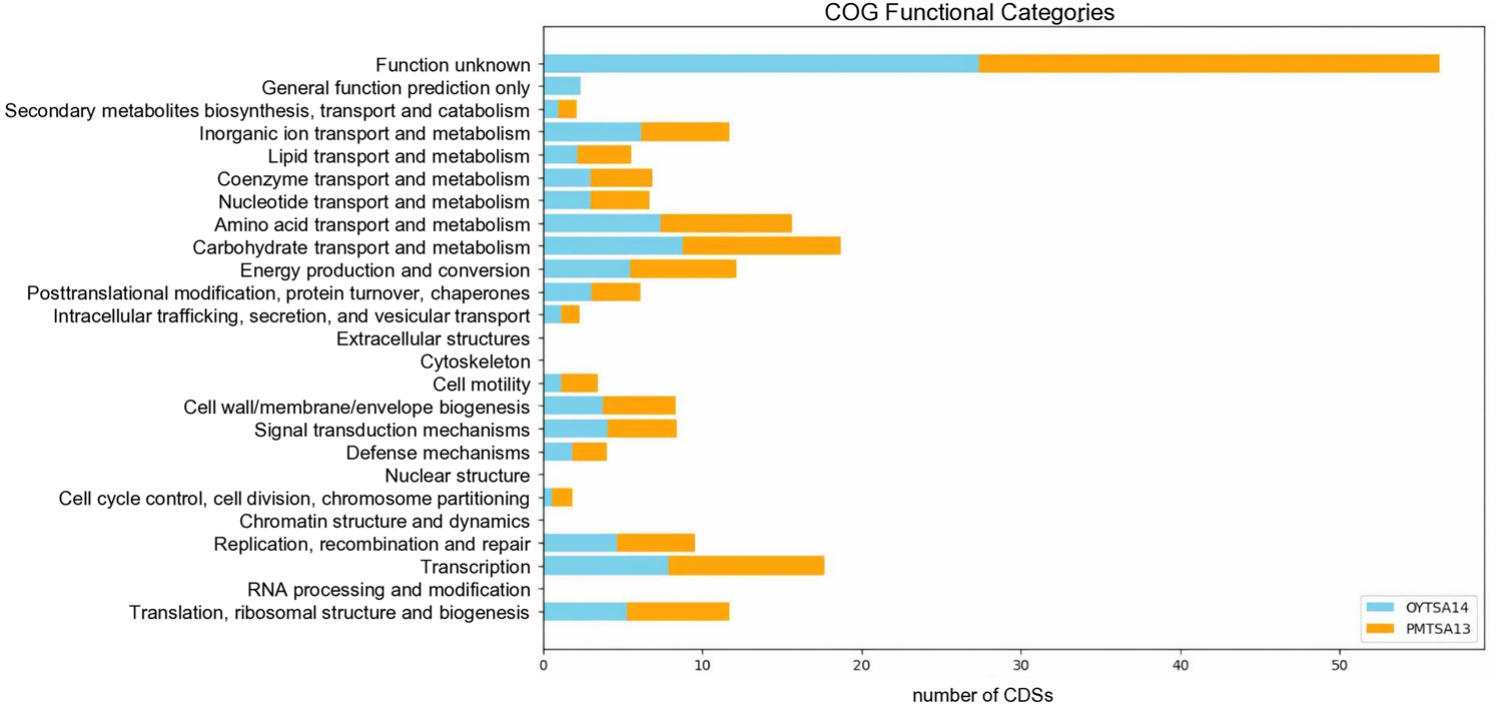
Analysis of the Cluster of Orthologous Groups (COG) within PMTSA13^T^ and OYTSA14. The distribution of genes in the whole genome sequence of strain PMTSA13^T^ and OYTSA14 was classified according to 25 eggNOG functional categories.

Cluster of Orthologous Group (COG) analysis using the eggNOG database showed the presence of total 2696 COG clusters in PMTSA13^T^ genome, and 2672 COG clusters in OYTSA14 genome, categorized into 24 functional categories (Fig. 3). The highest proportion of COG in these genomes is ‘unknown function’ (S) which constitute 27.39–28% of total COG, followed by ‘Carbohydrate transport and metabolism’ (G) which constitute 8.8–9.9% of total COG. No cytoskeletons (Z), extracellular (W), chromatin (B), and nuclear stuctures (Y) found within the genomes.

### Phenotypic characteristics

The PMTSA13^T^ and OYTSA14 strains were observed to form yellowish-white colonies, they are rod-shaped (0.4–0.5 µm wide and 1.1–1.4 µm long, Figs. S3 and S4), non-motile, facultatively anaerobic, catalase-positive, oxidase-negative, and Gram stain-positive. PMTSA13^T^ and OYTSA14 have an optimum growth condition at 30 °C and pH 7.0 on LB medium with the addition of 3% NaCl. Some of the enzyme and metabolic activities of PMTSA13^T^ and OYTSA14 were different from other reference strains, indicating that PMTSA13^T^ and OYTSA14 are distinct strains from the rest of the *Demequina* species (Table 1).

**Table 1:** Differential phenotypic characteristics of strain PMTSA13^T^ and OYTSA14 from closely related type strains.

Characteristic	1	2	3	4	5
Isolated from	Pepper root (rhizosphere)	Pepper root (rhizosphere)	Tidal flat sediment^a^	Mangrove soil^b^	Tidal flat sediment^c^
Temperature range for growth (°C)	4–40	10–37	25–37^a^	12–34^b^	5–35^c^
pH range for growth	5.0–9.0	5.0–9.0	6.0–8.0^a^	5.0–9.0^b^	6.0–11.0^c^
NaCl tolerance (%)	0–9	0–9	0-8^a^	0-8^b^	0-12^c^
Assimilation of (API 20NE)					
Esculin hydrolysis	+	+	+	+	+
l-arabinose	+	W	+	+	−
d-mannose	+	W	+	+	−
Mannitol	+	+	+	−	−
Maltose	+	+	+	+	−
Adipic acid	−	−	−	+	w
Malate	+	W	−	−	−
Trisodium citrate	+	+	+	−	+
Enzyme activity (API ZYM)					
Esterase (C4)	+	+	+	−	+
Esterase (C8)	W	W	+	−	W
Valine arylamidase	W	W	−	−	+
Acid phosphatase	−	+	W	−	−
α-Galactosidase	W	+	+	−	+

Strains: 1, PMTSA13^T^; 2, OYTSA14; 3, *Demequina zhanjiangensis* KCTC 52260^T^; 4, *Demequina salsinemoris* NBRC 105323^T^; 5, *Demequina aestuarii* NBRC 106155^T^. All data are based on observed conditions, except where indicated otherwise. +, positive; w, weakly positive; −, negative.

^a^Gao et al., 2023, ^b^Matsumoto et al., 2010, ^c^Yi et al., 2007.

### Chemotaxonomic features

The two strains were observed to contain two major fatty acids which are anteiso-C_15:0_ (40.78% and 47.89%, respectively) and C_16:0_ (12.89% and 9.45%), as detailed in Table 2. This composition is consistent across all referenced *Demequina* strains. The main differences from the closest related strain, *D. zhanjiangensis* SYSU T00b26 ^T^*,* are the presence of anteiso-C_16:0_ (2.15–2.39%), alcohol-C_16:0_ N (2.37–2.64%), and C_12:0_ (1.6–1.79%) in PMTSA13^T^ and OYTSA14 but not in *D. zhanjiangensis* SYSU T00b26 ^T^*.* Moreover, *D. salsinemoris* NBRC 105323^T^ shows the presence of iso-C_15:0_ (1.37%), and absence of iso-C_17:1_ *ω*5*c* and alcohol-C_16:0_ N. *D. aestuarii* NBRC 106155^T^ cell wall contain iso-C_17:0_ (1.39%) and iso-C_15:1_ G (1.38%) which different from all *Demequina.* These major fatty acids show that PMTSA13^T^ and OYTSA14 are distinct from other *Demequina* species, making them new species. Respiratory quinone of PMTSA13^T^ and OYTSA14 is demethylmenaquinone [DMK-9(H_4_)], which is the main characteristic of the genus *Demequina*^9–11^*.* Polar lipids observed in PMTSA13^T^ include diphosphatidylglycerol (DPG), phosphoglyceride (PG), phosphatidylinositol (PI), phosphatidylinositol mannosides (PIMs), and two unidentified lipid (L), while for OYTSA14 only DPG, PG, PIMs, and one L are observed (Fig. S5).

**Table 2:** Fatty acid composition of strain PMTSA13^T^, OYTSA14, and closely related species.

	1	2	3	4	5
Saturated					
C_12:0_	1.60	1.79	–	1.43	–
C_14:0_	8.84	8.93	3.76	**19.15**	2.23
C_16:0_	**12.89**	9.45	**11.46**	**11.48**	5.11
Unsaturated					
anteiso-C_17:1_ *ω*9*c*	–	–	–	–	1.31
iso-C_17:1_ *ω*5*c*	1.75	1.36	–	–	1.41
C_18:1_ *ω9c*	1.5	1.25	–	–	–
Branched					
iso-C_14:0_	3.09	3.00	1.40	5.15	1.64
iso-C_15:0_	–	–	2.98	1.37	3.37
iso-C_16:0_	5.95	5.45	4.68	4.22	6.45
iso-C_17:0_	–	–	–	–	1.39
anteiso-C_13:0_	3.18	2.96	1.00	2.2	1.83
anteiso-C_14:0_	1.57	1.47	–	1.00	–
anteiso-C_15:0_	**40.78**	**47.89**	**30.42**	**39.40**	**42.08**
anteiso-C_16:0_	2.39	2.15	–	1.17	1.96
Hydroxy					
iso-C_15:1_ G	–	–	–	–	1.38
C_14:0_ 2-OH	–	-	1.72	–	–
C_15:0_ 2-OH	1.78	1.22	-	2.86	1.58
C_16:0_ 2-OH	–	–	1.03	–	
anteiso-C_17:1_ A	1.76	1.38	–	–	–
alcohol-C_16:0_ N	2.64	2.37	–	–	1.93
Summed feature 3*	2.42	2.14	2.84	–	2.75
Summed feature 8*	2.42	2.14	22.66	1.26	–

Values are percentages of total fatty acid content. Data shown only for > 1% and major components (> 10%) are shown in bold. Strains: 1, PMTSA13^T^; 2, OYTSA14; 3, *Demequina zhanjiangensis* KCTC 52260^T^; 4, *Demequina salsinemoris* NBRC 105323^T^; 5, *Demequina aestuarii* NBRC 106155^T^. All data are based on observed conditions.

*Summed features are fatty acids that couldn’t be separated by GLC using the MIDI system. Summed feature 3 contains C_16:1_*ω*6*c* and/or C_16:1_*ω*7*c*; summed feature 8 contains C_18:1_*ω*7*c* and/or C_18:1_*ω*6*c*.

### Genomic insights into plant growth-promoting genes in PMTSA13^T^ and OYTSA14

Based on genome annotation and antiSMASH analysis, these two strains lack specific gene clusters or protein encoding genes associated with pathogen resistance or plant growth-promoting (PGP) activities (except tryptophan pathway) such as siderophore, antimicrobial, and nitrogen fixation. From antiSMASH analysis, only gene clusters for terpene was identified, consisting of two annotated regions. One of these regions exhibited 33% similarity with carotenoids, while the other did not display any similarity with other terpene clusters in the antiSMASH database. However, a significant portion of PMTSA13^T^ and OYTSA14 genes encode enzymes related to carbohydrate synthesis or metabolism. Research conducted by Kwak et al.^16^ suggests that bacteria possessing genomic features associated with carbohydrate metabolism and other functions may aid in adapting to life in the plant rhizosphere while also protecting the host plant against pathogens.

According to carbohydrate-active enzyme (CAZy) gene analysis using dbCAN3 (https://bcb.unl.edu/dbCAN2/)^17^, PMTSA13^T^ and OYTSA14 harbor 99 and 102 putative CAZy genes, respectively. These include 5 and 6 auxiliary activities (AA), 5 and 6 carbohydrate-binding modules (CBM), 3 and 4 carbohydrate esterases (CE), 58 and 60 glycoside hydrolases (GHs), 27 and 25 glycosyltransferases (GT), and 1 and 1 polysaccharide lyases (PL), respectively. There is a high proportion of glycoside hydrolases (GHs), which are enzymes capable of hydrolyzing the glycosidic bond between two carbohydrates^18^. Specifically, PMTSA13^T^ and OYTSA14 contain GH43 and GH127, which potentially encoding enzymes related to hemicellulose or pectin degradation. GH43 in *Bacillus licheniformis* has also been shown to trigger early defense responses in host plants and induce plant disease resistance, acting as a microbe-associated molecular pattern (MAMP)^19^. These two strains also contain a high portion of GT, which is mainly involved in biosynthesis of polysaccharides^20^.

Furthermore, these CAZymes not only break down complex substrates into simple sugars for the host’s use but also play a role in plant protection by hydrolyzing the cell walls of plant pathogens such as pathogenic fungi^21^. Genes in the GH family that encode antifungal properties, including chitinase (GH18), endoglucanase (GH51), and β-glucosidase (GH1), are all present in the genomes of PMTSA13^T^ and OYTSA14. To assess the functionality of these antifungal properties, we conducted experiments against various fungal pathogens. However, during 2 weeks of incubation, no antifungal activity was observed against *Rhizoctonia solani, Phytophthora cambivora, Botrytis cinerea*, *Colletotrichum acutatum*, *B. fabiopsis, Sclerotinia sclerotiorum,* and *P. capsici* (Fig. S6). Although the genome annotations of these strains indicate the presence of several genes that could encode enzymes with potential antifungal activity, our results do not confirm this activity. Several factors could explain this discrepancy, including incomplete gene pathways in the genome, inactivation of genes under the experimental conditions used, and species-specific antifungal properties that are ineffective against the pathogenic fungi tested in this experiment.

Previous studies has highlighted that genus *Demequina* played critical roles in metabolic, complex carbon degradation, and energy cycles in the aquatic ecosystem^13^. Annotation of PMTSA13^T^ and OYTSA14 genomes revealed the presence of several carbon-degrading enzymes, such as cellulase, cellobiose, arabinofuranosidase, chitinase, α-galactosidase, β-galactosidase, α-glucosidase, and β-glucosidase. The PMTSA13^T^ and OYTSA14 strains also contained genes involved to riboflavin biosynthesis, including *ribABDEH*, known to promote plant growth due to its significance in several primary metabolic pathways in plants^22^. Additionally, riboflavin plays a role in plant growth by compensating for oxidative burst effects and inducing systemic resistance^23^. Moreover, these strains possess genes associated with sulfur metabolism, specifically *cysCDHNQ,* which can facilitate the breakdown of sulfur into sulfate, potentially enhancing sulfate availability in the plant medium. The strains also contain genes encoding proteins implicated in indole-3-acetic acid (IAA) biosynthesis, including *trpABCDEF,* which may contribute to their plant growth-promoting abilities. These findings highlight the diverse metabolic capabilities of PMTSA13^T^ and OYTSA14 and their potential significance in plant–microbe interactions.

### Plant growth-promoting activity of PMTSA13^T^ and OYTSA14

Several enzymatic activities associated with PGP traits were examined in PMTSA13^T^ and OYTSA14. These strains are able to breakdown cellulose to glucose, which is crucial to help the availability of simple sugars for plants nutrient uptake (Fig. S7). These strains also exhibited catalase activity, which plays a vital role in degrading H_2_O_2_ and alleviate the effect of oxidative stress in plants^24^. However, these strains showed negative results for siderophore production, amylase, gelatinase, phosphate solubilization, protease, and nitrogen fixation activities (Table S1).

**Figure 4:**
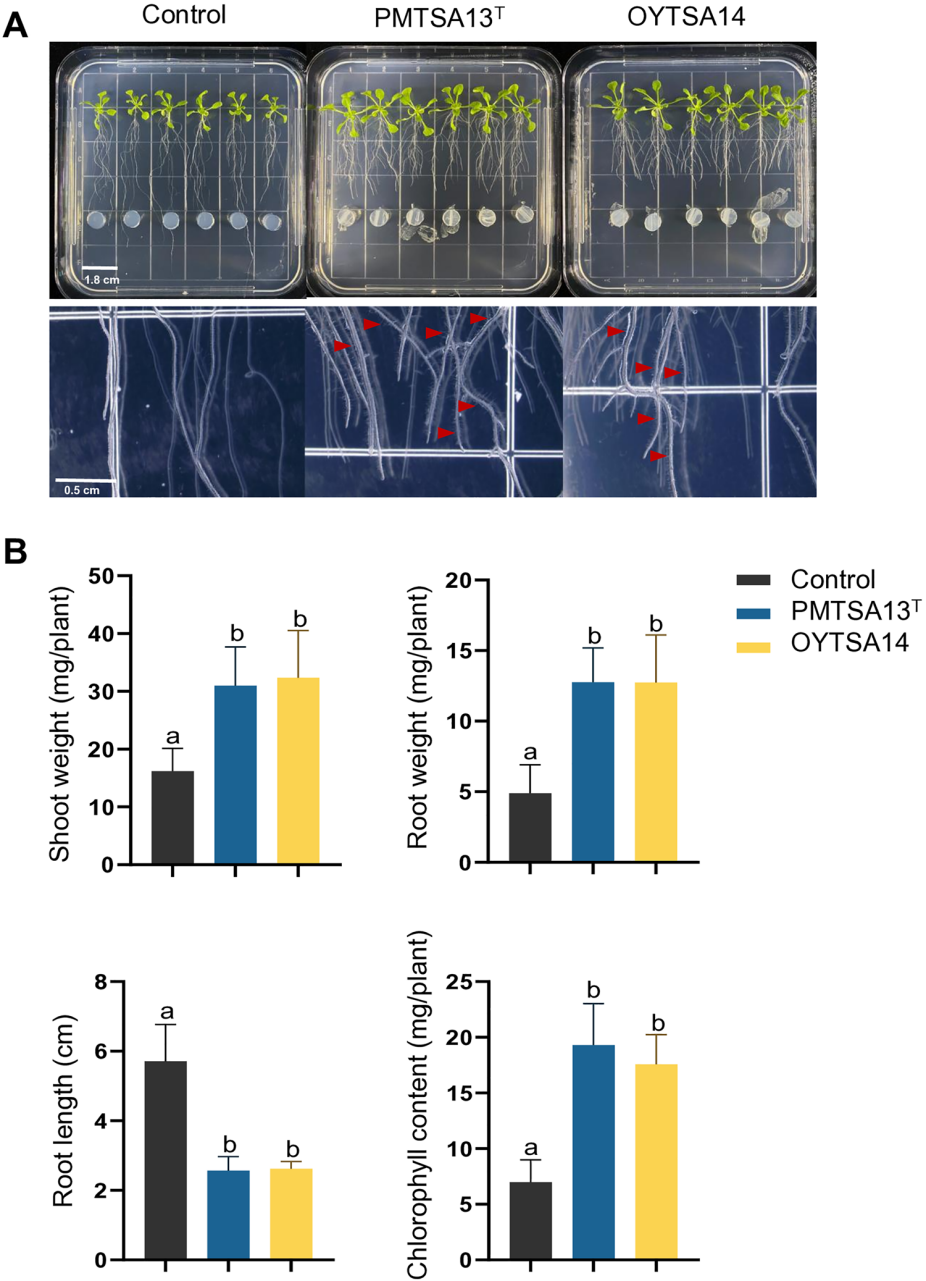
Effects of PMTSA13^T^ and OYTSA14 on the growth of *Arabidopsis thaliana* seedlings. (**A**) Growth of *A. thaliana* in 1/2 MS medium for 10 days at 25 °C. Plants were co-inoculated with PMTSA13^T^ and OYTSA14, alongside a control group without any inoculation. Root hairs are highlighted by red arrows. (**B**) Quantification of shoot weight, root weigth, root length, and chlorophyll contents. Error bars indicate the standard deviation of the mean (*n* = 6). Letters (a, b) indicate a statistically significant difference between the control and the bacterial inoculation (one-way ANOVA, *p* < 0.05). Experiments were repeated twice with similar results.

**Figure 5:**
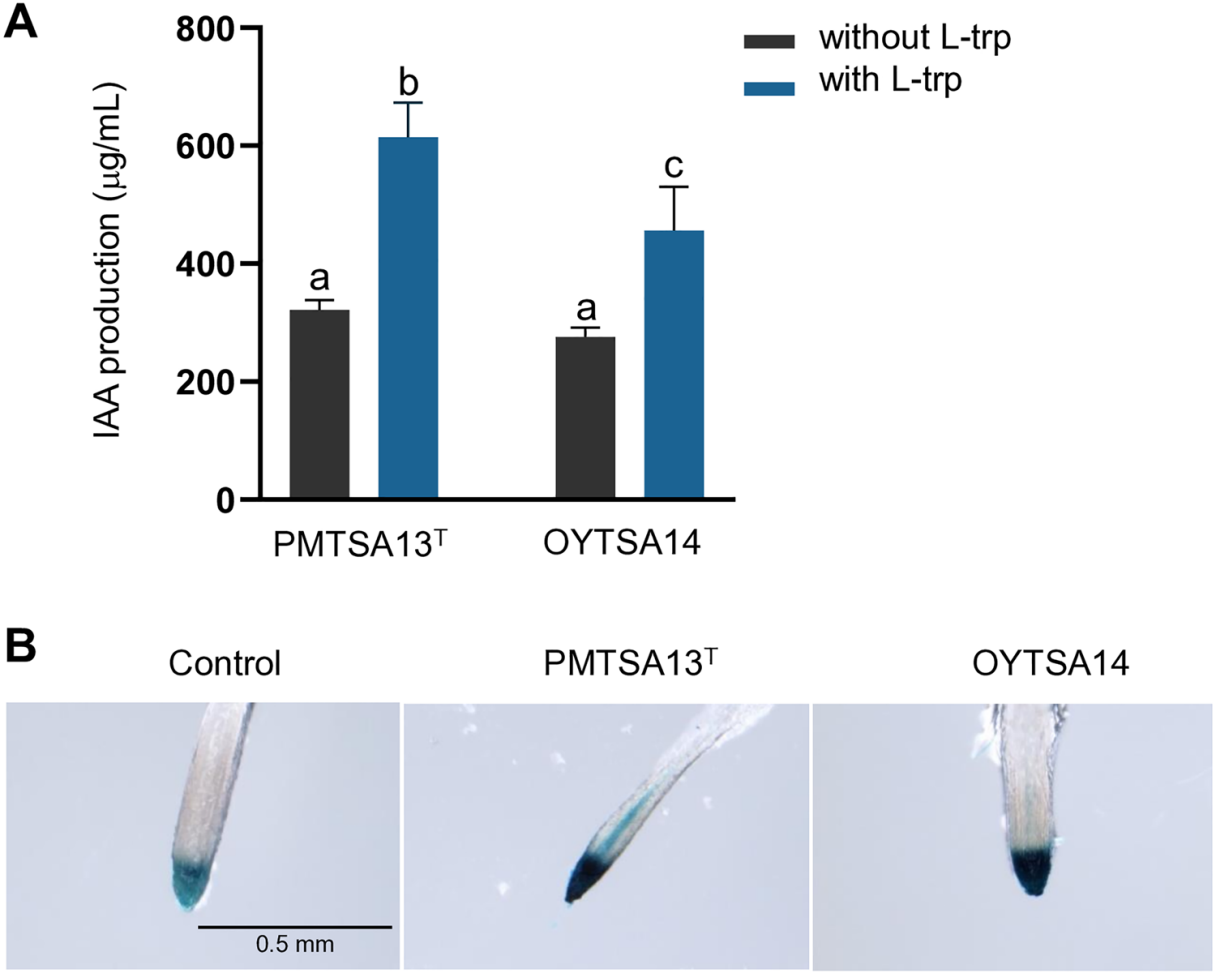
Production of Indole-3-acetic acid (IAA) by PMTSA13^T^ and OYTSA14, and their effect in the DR5::GUS *Arabidopsis* seedling roots. (**A**) Graph showing IAA production levels by PMTSA13^T^ and OYTSA14 with and without l-tryptophan supplementation. (**B**) Visualization of GUS staining in the DR5::GUS *Arabdopsis* seedling roots, where blue coloration signifies the expression of DR5.

To evaluate the effects of PMTSA13^T^ and OYTSA14 on plant development, transgenic *Arabidopsis* line DR5::GUS seedlings were exposed to these bacteria in 1/2 MS medium for 10 days at 25 °C (Fig. 4). Shoot weight, root weight and length, and chlorophyll content were measured. PMTSA13^T^ and OYTSA14 significantly promoted plant growth by increasing shoot weight 1.9-fold to twofold, root weight 2.6-fold, and chlorophyll contents 2.5-fold to 2.8-fold compared to the control (Fig. 4B). These strains also stimulated the development of root branching and root hairs, facilitating enhanced nutrient uptake from the soil^25^ (Fig. 4A). Moreover, IAA production by these strains was analyzed by adding and without tryptophan as a precursor in liquid LB grow medium. PMTSA13^T^ produced IAA up to 615 μg/mL with the addition of tryptophan and 322 μg/mL without tryptophan, while OYTSA14 produced 457 μg/mL and 276 μg/mL, respectively (Fig. 5A). Expression level of DR5::GUS promoter after staining was also observed following inoculation with PMTSA13^T^ and OYTSA14, with expression levels higher than the control. This indicates a higher level of auxin signaling in *Arabidopsis* DR5::GUS seedlings after inoculation with these strains, as showed by a more intense blue color in the roots (Fig. 5B). These data suggests that PMTSA13^T^ and OYTSA14 promote plant growth by producing auxin hormone and affecting auxin signaling in plants.

## Conclusion

Strains PMTSA13^T^ and OYTSA14, isolated from the rhizosphere of *Capsicum annuum*, were designated as novel species within the genus *Demequina* based on phylogenetic, phenotypic, and chemotaxonomic analyses. Genome annotation revealed the presence of several plant growth-promoting traits, including indole-3-acetic acid (IAA) production, catalase and cellulose activity, sulfur metabolism, riboflavin biosynthesis, various carbon-degrading enzymes, and a high carbohydrate transport and metabolism COG. This was further confirmed by direct inoculation of PMTSA13^T^ and OYTSA14 onto *A. thaliana*, which exhibited significant plant growth-promoting activity, resulting in up to a twofold increase in growth compared to the control and higher expression of DR5::GUS promoter.

Description of *Demequina capsici* sp. nov.

*Demequina capsici* (cap’si.ci. N.L. gen. neut. n. capsici, referring to *Capsicum*, the genus name of pepper).

Colonies grown on LB medium were observed as convex, circular, and yellowish-white colour colony with a size of 0.4–0.5 µm wide and 1.1–1.4 µm long after 2 days of incubation on LB agar at 30 °C. Cells are gram-positive, rod-shaped, non-motile, and facultatively anaerobic. These strains are capable to grow on LB, TSA, R2A, MA, YEA , but not on PDA, showing optimal growth on LB medium. PMTSA13^T^ can grow weakly in temperature range of 4–15 °C and 40 °C, meanwhile, OYTSA14 from 10 to 37 °C, with optimum growth for both strains at 30 °C. These strains can tolerate NaCl additions in growth media up to 9% (w/v) with optimum growth at 3% NaCl, and the pH range for growth is 5.0–9.0 with optimum pH at 7.0. Catalase positive and oxidase negative. API 20NE results show that only esculine hydrolysis and activity of β-galactosidase are observed, also positive for assimilation of d-glucose, l-arabinose, d-mannose, mannitol, maltose, trisodium citrate, and weakly for malate. These strains could utilize l-arabinose, d-ribose, d-xylose, d-galactose, d-glucose, d-fructose, d-mannose, l-rhamnose, d-mannitol, methyl-αd-mannopyranoside, methyl-αd-glucopyranoside, *N*-acetylglucosamine, amygdalin, arbutin, esculin ferric citrate, salicin, d-cellobiose, d-maltose, d-lactose (bovine origin), d-melibiose, d-saccharose (sucrose), d-trehalose, d-raffinose, amidon (starch), glycogen, gentiobiose, d-turanose, d-lyxose, potassium gluconate, and potassium 5-KetoGluconate. PMTSA13^T^ and OYTSA14 showed activity for esterase (C4), esterase (C8), lipase (C14), leucine arylamidase, valine arylamidase, cystine arylamidase, trypsin, α-chymotrypsin, acid phosphatase, naphthol-AS-BI-phosphohydrolase, α-galactosidase, β-galactosidase, β-glucuronidase, α-glucosidase, and β-glucosidase enzymes. The DNA G+C content of PMTSA13^T^ is 69.8% and 69.7% for OYTSA14. The dominant fatty acids are anteiso-C_15:0_ and C_16:0_, while major polar lipids are DPG, PG, and PIMs. The predominant ubiquinone is DMK-9(H_4_).

The GenBank accession number of 16S rRNA sequences of PMTSA13^T^ and OYTSA14 is OR632336.1 and OR632335.1, respectively, while whole genome sequences are CP134880.1 and CP134879.1. These strains are available in Korea Collection for Type Culture (KCTC 59028^T^) and Guangdong Microbial Culture Collection Center (GDMCC 1.4451^T^).

## Materials and methods

### Sample isolation and culture conditions

PMTSA13^T^ and OYTSA14 were isolated from the rhizosphere of *Capsicum annuum* at the Jeongeup plant nursery (35.5638997 N, 126.8540235 E), South Korea. Rhizosperic samples were obtained by mixing 1 g of soil from the sampling site with sterilized 1× PBS. The solution of this mixture was then diluted and spread onto Reasoner’s 2 Agar (R2A) (Difco), and the bacteria were allowed to grow for 3 days at 25 °C. Growing colonies were then restreaked to obtain single colonies, and colonies with round, yellowish-white appearance were isolated from the medium and designated as PMTSA13 and OYTSA14. These strains were preserved in 10% skim milk at − 80 °C for further analysis. The strains were deposited in Korean Collection for Type Cultures (PMTSA13^T^ = KCTC 59028^T^, OYTSA13 = KCTC 59027) and Guangdong Microbial Culture Collection Center (PMTSA13^T^ = GDMCC 1.4451^T^, OYTSA14 = GDMCC 1.4450).

### Phylogenetic analysis

The 16S rRNA gene of PMTSA13^T^ and OYTSA14 was amplified using extracted genomic DNA as the template, and the primer pair 27F and 1492R was used for PCR amplification. Subsequently, the PCR products were sequenced using the universal primer pair 518F and 805R^26^. The sequencing was conducted by BIOFACT company in Daejeon, Repulic of Korea, and the resulting sequences were assembled using Vector NTI software (1.6.1). The almost complete sequences of 16S rRNA sequences (1455 bp for PMTSA13^T^ and 1457 bp for OYTSA14) were compared with the closest related sequences using pairwise similarity values in the EzBiocloud server (http://www.ezbiocloud.net/)^27^ and the GenBank database using the BLAST algorithm (https://blast.ncbi.nlm.nih.gov/Blast.cgi), identifying 24 closely related species withinin the genus *Demequina*. The 16S rRNA sequences of these closely related species, with *Rathayibacter rubneri* ZW T219 as an outgroup, were then aligned using ClustalW for Multiple Sequence Alignment^27^. A phylogenetic tree employing neighbour-joining (NJ), minimum-evolution (ME), and maximum likelihood (ML) algorithms algorithms was then constructed using the Molecular Evolutionary Genetics Analysis (MEGA 7.0) software^28^, with 1000 bootstrap iterations.

### Genome sequencing and assembly

Genomic DNA from PMTSA13^T^ and OYTSA14 was extracted using Genomic DNA extraction kit (MGmed, Republic of Korea) according to the manufacturer’s protocol, and subjected to whole-genome sequencing at Macrogen Co. Ltd. using the PacBio Sequel II system (Pacific Biosciences, Inc.) and the Illumina sequencing platform. The genomes were assembled de novo using the SMRT Portal’s Microbial Genome Assembely application (version 2.3), with Illumina reads subsequently used to refine the assembly accuracy via Pilon (version 1.21)^29^. To assess the completeness of assembled genomes, BUSCO (Benchmarking Universal Single-Copy Orthologs) analysis was performed^30^. Whole genome sequences of PMTSA13^T^ and OYTSA14 were then deposited in the GenBank database, accompanied by annotation generated through the Prokaryotic Genome Annotation Pipeline (PGAP)^31^.

### Comparative genomics and genome features

For comparative analysis, genome sequences of 24 *Demequina* species were retrieved from the NCBI and NMDC database. Further taxonomic classification was performed using the Orthologous ANI Tool online (OAT, version 0.93.1)^32^ and Genome-to-Genome Distance Calculator 3.0 (GGDC) available at https://ggdc.dsmz.de/ggdc.php^33^. Gene prediction was conducted using Prokka (1.14.6)^34^, while genome annotation was facilitated by BLAST+^35^. Gene function characterization was performed with BlastKOALA. The eggNOG database ^36^ was employed to identify Cluster of Orthologous Group (COG) gens. The dbCAN3 meta server at https://bcb.unl.edu/dbCAN2/ was used for identifying carbohydrate-active enzyme (CAZy) genes^17^. Additionally, the AntiSMASH tool was utilized for the prediction of gene clusters within bacteria genomes^37^.

### Phenotypic and biochemical characteristics

To investigate the physiology and chemotaxonomy, *Demequina salsinemoris* NBRC 105323^T^, *Demequina zhanjiangensis* KCTC 52260^T^, and *Demequina aestuarii* NBRC 106155^T^ were acquired from collection center and cultured in specific media for comparative studies. The growth parameters of strains PMTSA13^T^ and OYTSA14 were evaluated by subjecting them to various conditions. Cultivation was conducted on different agar media, including Luria–Bertani (LB) agar, tryptic soy agar (TSA), Reasoner’s 2A agar (R2A), marine agar (MA), yeast extract agar (YEA), and potato dextrose agar (PDA) at 30 °C for 2 days. Additionally, the bacteria were incubated on LB at temperatures from 4, 10, 15, 20, 25, 30, 35, 37, 40, 45, 50, 55, and 60 °C for 5 days to assess the impact of different temperatures. Varied pH levels (ranging from 3.0 to 12.0 with intervals of 1.0), a range of NaCl concentrations (0–12% (w/v) NaCl at 1.0% intervals), and anaerobic conditions using oxygen-free incubator were also considered in this analysis.

Gram staining was performed on 2-day cultures of each strain using the Gram Stain Solution kit (Difco) in accordance with the manufacturer’s instructions. Bacterial motility was analyzed by culturing bacteria in LB with 0.4% agar for 3 days^38^. Catalase activity was determined by bubble production after adding 3% (v/v) hydrogen peroxide, and oxidase activity was analyzed with 1.0% tetramethyl-*p*-phenylenediamine (bioMérieux). Furthermore, cell morphology was examined with scanning electron microscopy (SEM) and transmission electron microscopy (TEM). Biochemical characteristics were assessed using API ZYM, API 20NE, and API 50CH kits (bioMérieux) following the manufacturer’s instructions.

### Chemotaxonomy features

Chemotaxonomy analysis included the examination of polar lipid, quinone, and fatty acid. Bacteria were grown in 1 L TSB flasks at 30 °C with shaking at 130 rpm for 5 days, after which cells were collected by centrifugation at 12,000 rpm for 15 min. For polar lipids analysis, lipids were extracted and then analyzed using two-dimensional thin liquid chromatography (TLC)^39^, using the first solvent mixture of chloroform, methanol, and distilled water (65:25:4, v/v) and a second solvent mixture of chloroform, acetic acid, methanol, and distilled water (80:15:12:3, v/v). TLC plates was visualized using various reagents including Dragendorff solution (Sigma-Aldrich), α-naphthol, molybdenum blue (Sigma-Aldrich), phosphomolybdic acid (Sigma-Aldrich), and 0.2% ninhydrin (Sigma-Aldrich). Respiratory quinone was extracted following the method described by Collins et al.^40^ and analyzed using high-performance liquid chromatography (HPLC) with UV absorption at 270 nm wavelength using a methanol and isopropanol (7:5, v/v) mobile. For fatty acids analysis, bacteria cells were scraped from the culture plate, saponified, methylated, and then extracted according to the procedure outline by Sasser^41^. Extracted fatty acids were subsequently analyzed using gas chromatography.

### Plant growth-promoting traits

The production of indole-3-acetic acid (IAA) was assessed by culturing PMTSA13^T^ and OYTSA14 in 10 mL LB medium, with and without 0.1% L-Tryptophan, followed by incubation at 30 °C with agitation at 130 rpm for 5 days. After incubation, cells were centrifuged, and 500 μl of supernatant was mixed with an equal volume of Salkowski reagent (0.5 M FeCl_3_ in a solution of water and concentrated H_2_SO_4_ in a 1:50:30 ratio, v/v). This mixture was then incubated in the dark for 30 min, and the absorbance was measured at 530 nm using a UV–Vis microplate spectrophotometer (Thermofisher, US). Siderophore production of PMTSA13^T^ and OYTSA14 was determined by placing agar disks from cultures on CAS agar and incubating for 7 days^42^. The activities of cellulase, amylase, protease, and gelatinase were assessed by incubating the strains on respective agar plates for 3 days;Cellulase activity on LB agar supplemented with 1% carboxymethylcellulose (CMC) and stained with 0.1% Congo red.;amylase activity was assessed on LB agar with 1% soluble starch, visualized by adding iodine; protease activity on a medium containing tryptone, skim milk powder, yeast extract, and agar (5:25:3:12 w/w); and gelatinase on LB agar with 1% gelatin. Phosphate solubilization activity was evaluated using Pikovskaya’s medium (PVK) after initial growth on LB agar for 2 days, with culture discs then transferred to PVK medium for a 5 days incubation^43^. Nitrogen fixation ability was tested by applying 10 μl drops of liquid culture onto Jensen’s medium, followed by incubation for 5 days.

The antifungal activity assay was conducted through *in-vitro* experiments against several crop fungal pathogens. The pathogens used, obtained from the Korea Agricultural Culture Collection (KACC), were *Phytophthora cambivora* KACC 40160, *Sclerotinia sclerotiorum* KACC 41065, *Botrytis fabiopsis* KACC 40962, *Rhizoctonia solani* AG-1 KACC 40101, *Botrytis cinerea* KACC 40573, *Colletotrichum acutatum* KCTC 40804, *Phytophthora capsici* KACC 47699, and *Phytophthora capsici* KACC 47698. The bacterial strains PMTSA13^T^ and OYTSA14 were grown on LB agar plates for 2 days at 30 °C. Agar blocks containing bacterial cultures were then placed onto PDA plates that contained block of the pathogenic fungi culture. The plates were incubated at 37 °C for 2 weeks. Antifungal activity was assessed by observing inhibition zones around the bacterial blocks. Plant growth-promoting experiments were conducted by co-inoculating PMTSA13^T^ and OYTSA14 with auxin-reporter system DR5::GUS in *Arabidopsis* seedlings. The transgenic *Arabidopsis* line DR5::GUS^44^ seeds were surface sterilized and subjected to a 2 days cold treatment at 4 °C to break dormancy, followed by germination on Murashige and Skoog (MS) media and allowed to germinate for 7 days at 22 °C. Germinated seedlings were then transferred to 1/2 MS media, with discs of cultured PMTSA13^T^, OYTSA14, and blank (LB agar only) added to the 1/2 MS media approximately 3.6 cm from the root tip. The growth of *A. thaliana* DR5::GUS was monitored for 10 days at 22 °C under a 16-h light/8-h dark cycle. Each experiment (control, PMTSA13^T^, OYTSA14) utilized 3 plates and was repeated twice. Root morphology, production of IAA, shoot weight, root weight, root length, and chlorophyll content were observed and measured. Expression of DR5::GUS in seedling was visualized using GUS staining, as described by Jefferson et al.^45^, and observed under a microscope.

### Ethics approval

Experimental research and field studies on plants, including the collection of plant material, complying with relevant institutional, national, and international guidelines and legislation.

### Supplementary Information

Supplementary Information.

## Data Availability

Strain PMTSA13^T^ and OYTSA14 can be obtained from Korean Collection for Type Cultures (PMTSA13^T^ = KCTC 59028^T^, OYTSA13 = KCTC 59027) and Guangdong Microbial Culture Collection Center (PMTSA13^T^ = GDMCC 1.4451^T^, OYTSA14 = GDMCC 1.4450). The 16S rRNA gene sequence of strain PMTSA13^T^ is available under GenBank accession number OR632336.1, while the whole-genome sequence is CP134880.1. For OYTSA14, the 16S rRNA gene sequence is available under GenBank accession number OR632335.1, and CP134879.1 for whole genome sequence. The associated BioSample and BioProject accession numbers are SAMN23170019 and PRJNA678113, respectively. The taxonomy ID for strain PMTSA13^T^ is 3075620, while OYTSA14 is 3075619.
