# Supplementary material for: Demequina capsici sp. nov., a novel plant growth-promoting actinomycete isolated from the rhizosphere of bell pepper (Capsicum annuum)
Source: Sci Rep. 2024 Jul 9;14:15830. doi: 10.1038/s41598-024-66202-x (PMC11233565; doi:10.1038/s41598-024-66202-x)
Supplement: Supplementary file 1 — Supplementary Information. [file 41598_2024_66202_MOESM1_ESM.pdf]

Supplementary Information

Demequina capsici sp. nov., a novel plant growth-promoting actinomycete isolated from the rhizosphere of bell pepper (*Capsicum annuum*)

Zalfa Humaira[1,2], Donghyun Cho[1], Yuxin Peng[1], Forbes Avila[3,4], Yu Lim Park[1], Cha Young Kim[1], Jiyoung Lee[1, 2*]

[1]Korean Collection for Type Cultures (KCTC), Biological Resource Center, Korea Research Institute of Bioscience & Biotechnology (KRIBB), Jeongeup, Jeollabuk-do 56212, Republic of Korea

[2]Department of Biosystems and Bioengineering, KRIBB School of Biotechnology, University of Science and Technology (UST), Yuseong, Daejeon 34113, Republic of Korea

[3]Animal Model Research Group, Jeonbuk Branch Institute, Korea Institute of Toxicology, Jeongeup, Jeollabuk-do 56212, Republic of Korea

[4]Human and Environmental Toxicology, Korea National University of Science and Technology (UST), Yuseong, Daejeon 34113, Republic of Korea

[*] Corresponding author: J.Lee; jiyoung1@kribb.re.kr

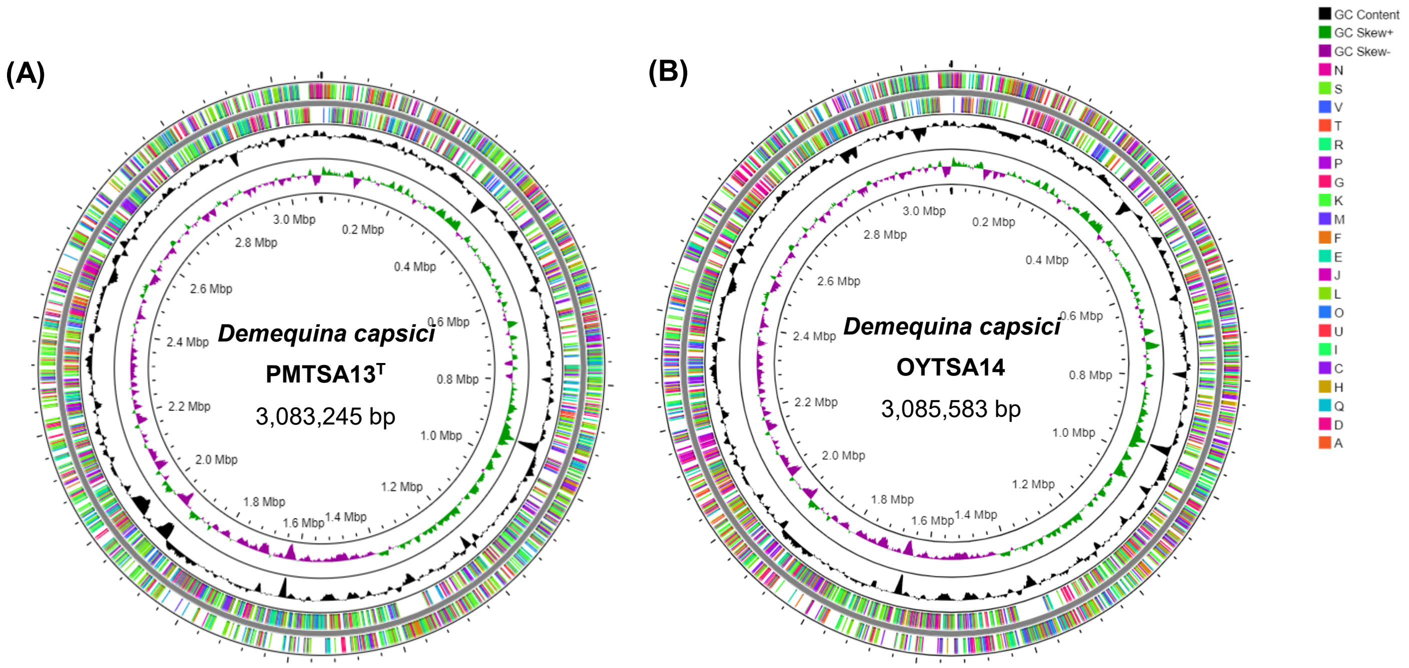

Figure S1. Genome maps of the PMTSA13T (A) and OYTSA14 (B) strains, generated with Proksee, are displayed. Annotation of genome features is depicted from outer to inner rings. Ring 1 and 2 show annotation of Cluster of Orthologous Group (COG) in the forward and reverse directions, respectively. Ring 3 illustrates GC content plots, while ring 4 displays GS skew plots. COG categories include: A, RNA processing and modification; B, chromatin structure and dynamics; C, energy production and conversion; D, cell cycle control, cell division, chromosome partitioning; E, amino acid transport and metabolism; F, nucleotide transport and metabolism; G, carbohydrate transport and metabolism; H, coenzyme transport and metabolism; I, lipid transport and metabolism; J, translation, ribosomal structure and biogenesis; K, transcription; L, replication, recombination, and repair; M, cell wall/membrane/envelope biogenesis; N, cell motility; O, posttranslational modification, protein turnover, chaperones; P, inorganic ion transport and metabolism; Q, secondary metabolites biosynthesis, transport, and catabolism; R, general function prediction only; S, function unknown; T, signal transduction mechanisms; U, intracellular trafficking, secretion, and vesicular transport; V, defense mechanisms.

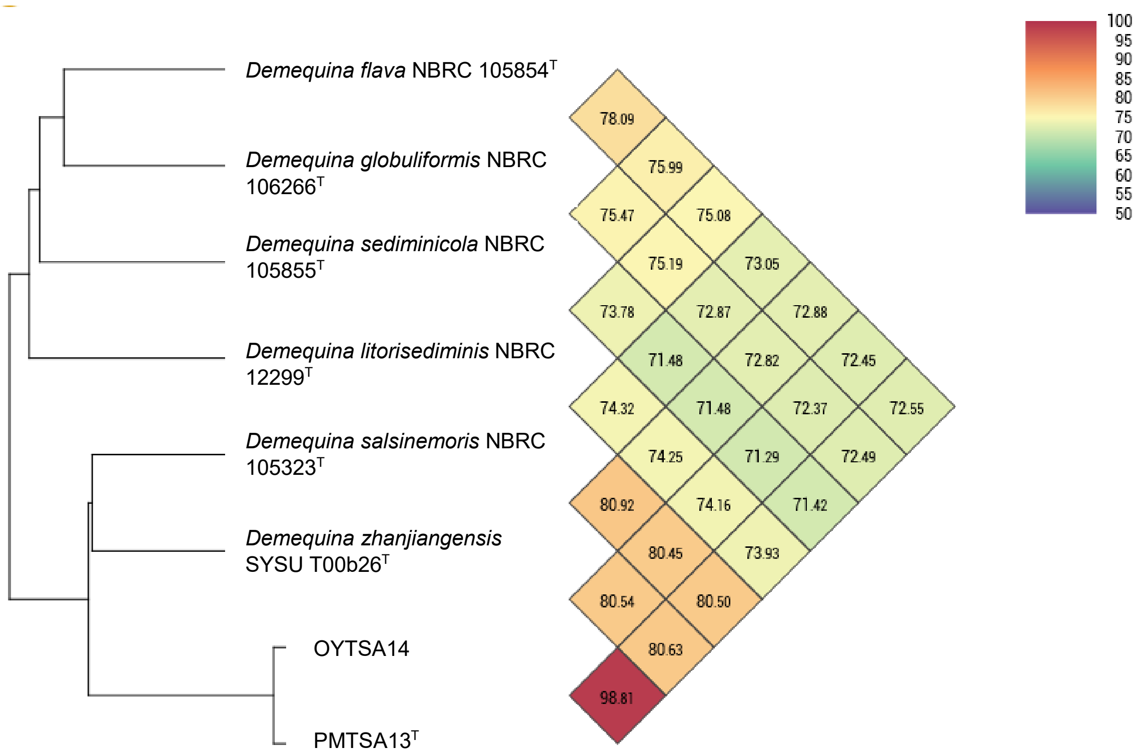

Figure S2. OrthoANI heatmap generated using OAT software (version 0.93.1) between PMTSA13^T, OYTSA14, and other closely related taxa of genus *Demequina*. Value of OrthoANI below 96% indicates that the strains belong to different species.

(A)

(B)

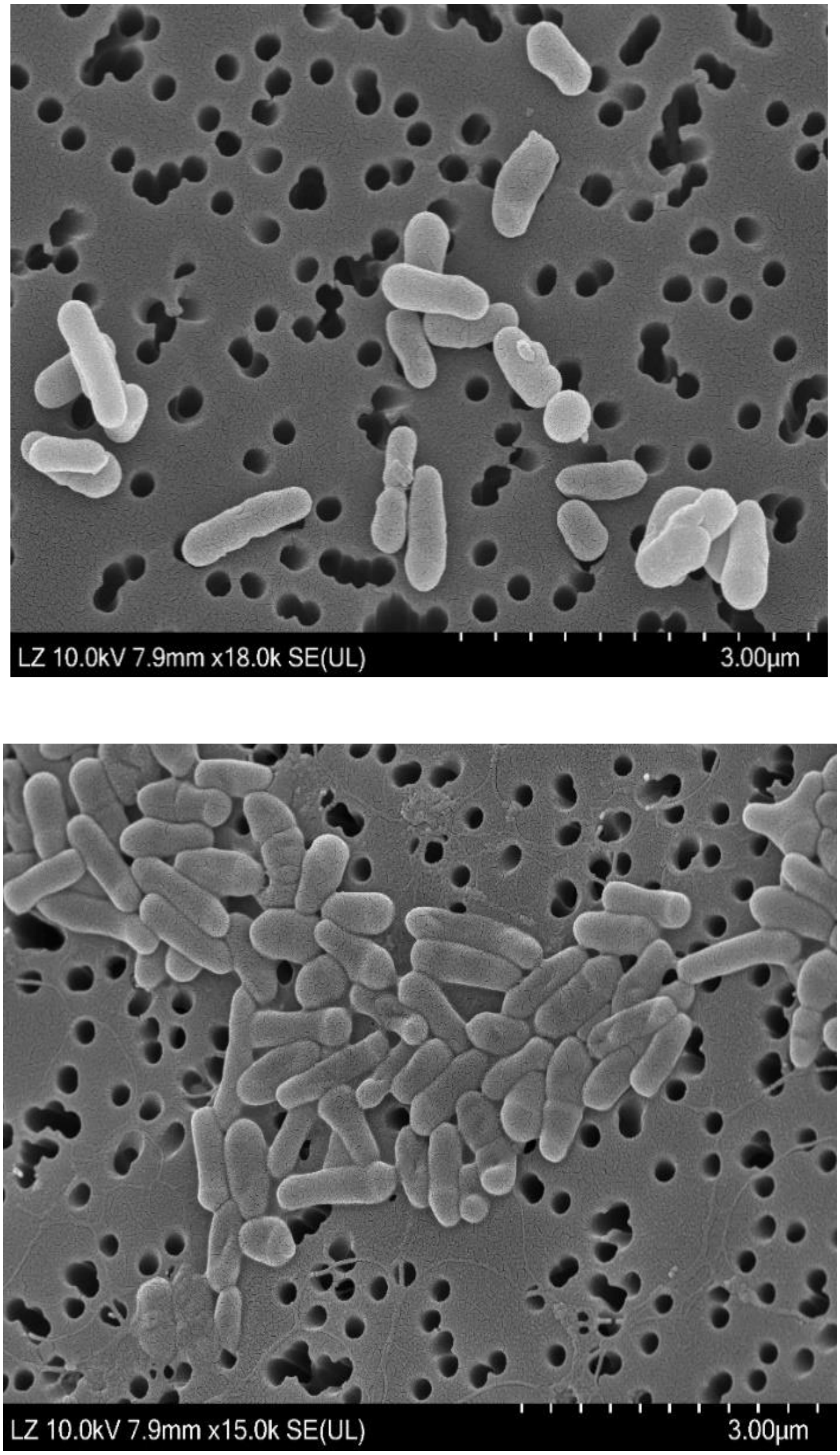

Figure S3. Scanning electron microscopy of strains PMTSA13^{T} (A) and OYTSA14 (B) after 3 days growth on R2A medium at 30 °C. Bars, 3.00 μm.

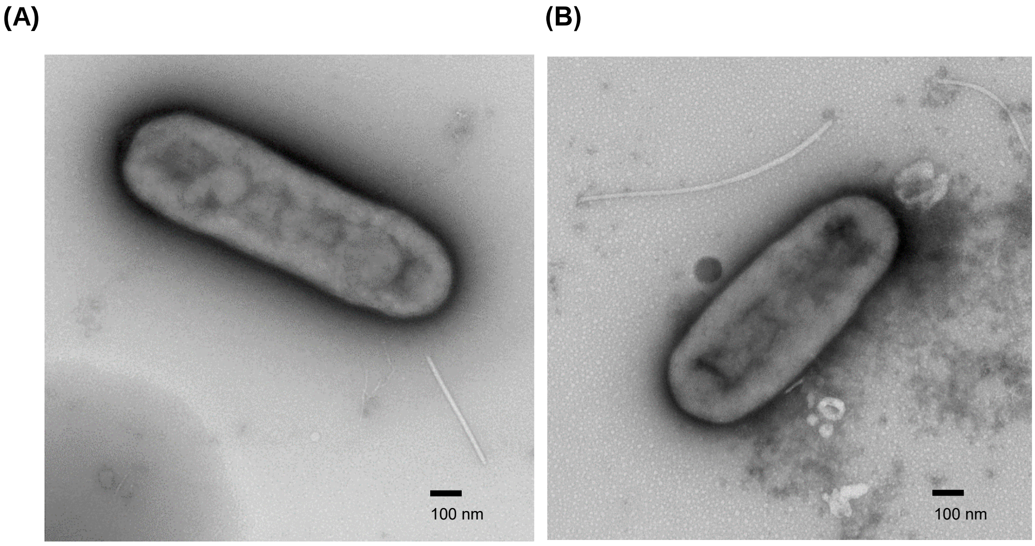

Figure S4. Transmission electron microscopy of strain PMTSA13^{T}(A) and OYTSA14(B) after 3 days growth on R2A medium at 30 °C. Scale bar, 100 nm.

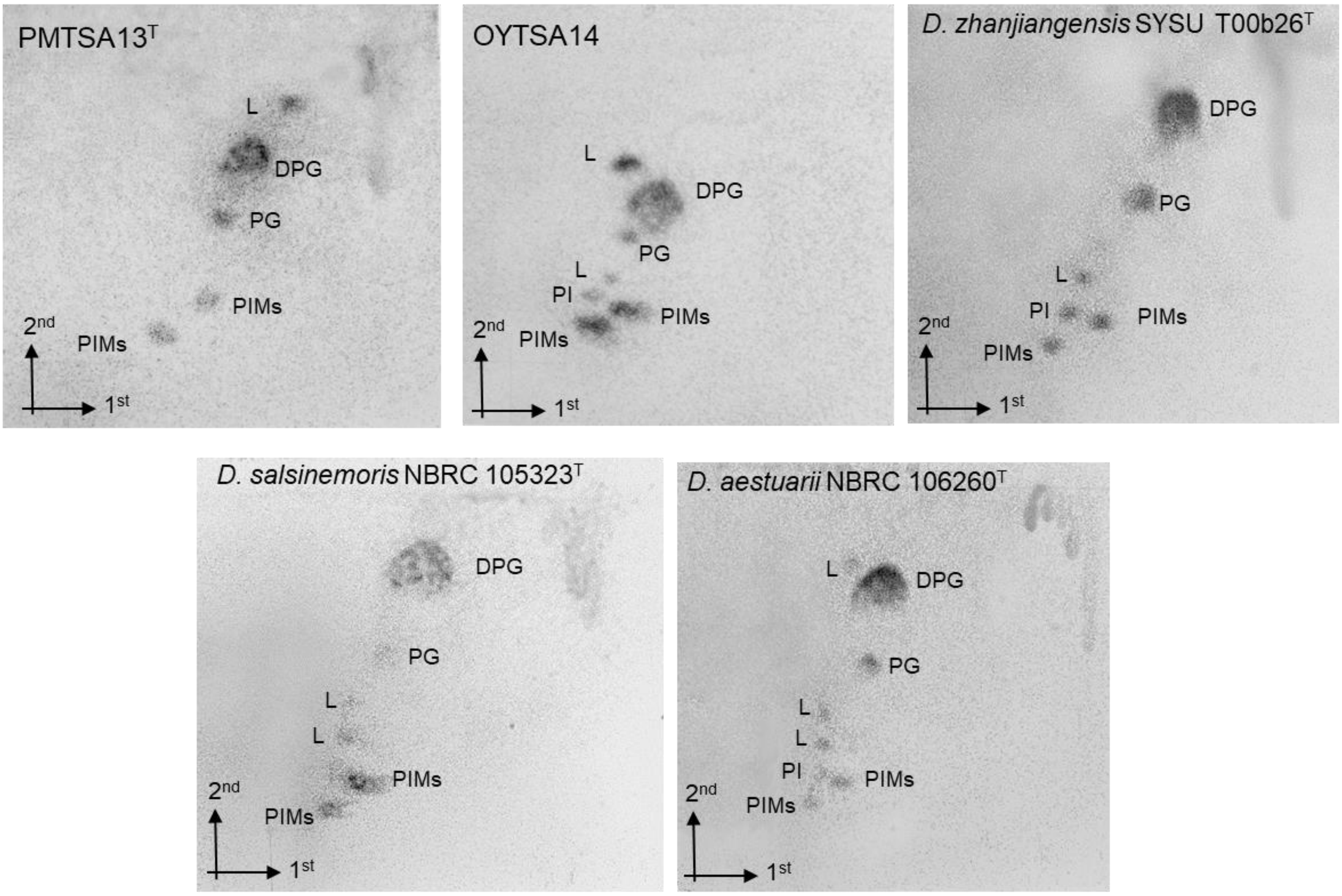

Fig. S5. Two-dimensional thin-layer chromatography of polar lipids of strains PMTSA13^{T} and OYTSA14 following staining with Dragendorff reagent, 0.2% ninhydrin, α-naphthol, molybdenum blue, and phosphomolybdic acid. Diphosphatidylglycerol (DPG), phosphoglyceride (PG), phosphatidylinositol (PI), phosphatidylinositol mannosides (PIMs), and unidentified lipid (L) were observed.

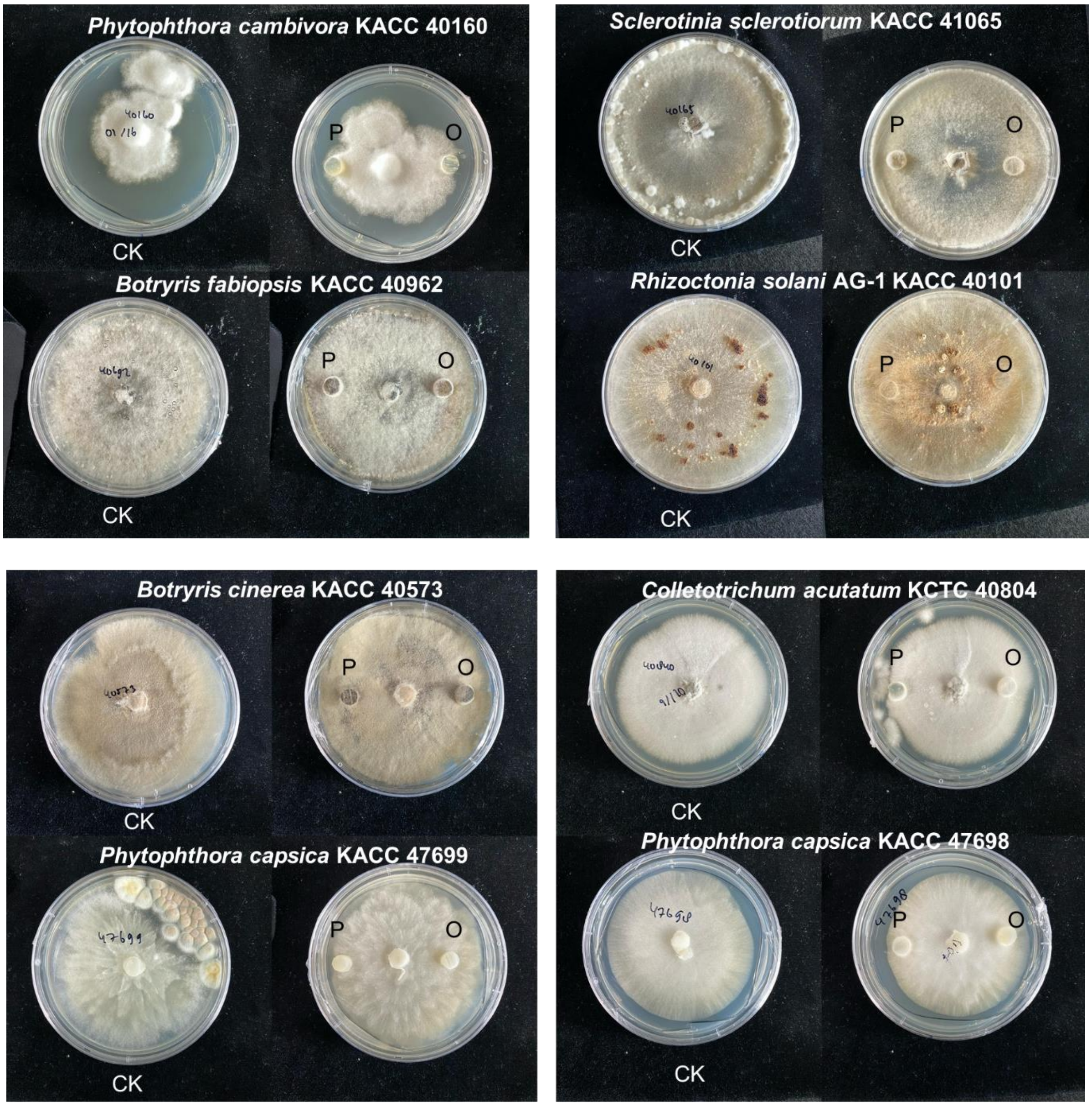

Figure S6. Antifungal experiments conducted using PMTSA13^{T} and OYTSA14 against various fungal pathogens, including *Phytophthora cambivora* KACC 40160*, Sclerotinia sclerotiorum* KACC 41065*, Botryris fabiopsis* KACC 40962*, Rhizoctonia solani* AG-1 KACC 40101*, Botryris cinerea* KACC 40573*, Colletotrichum acutatum* KCTC 40804*, Phytophthora capsica* KACC 47699*, Phytophthora capsica* KACC 47698*.* The experiment was carried out over a period of 2 weeks on potato dextrose agar (PDA). Agar blocks containing 2-day-old cultures of PMTSA13^{T} (P) and OYTSA14 (O) were used, as these strains could not grow directly on PDA.

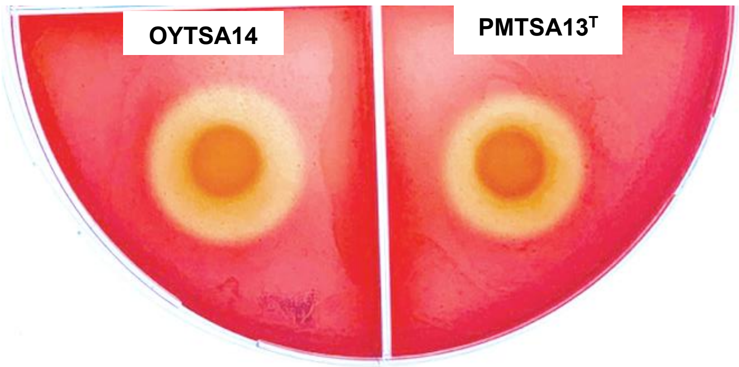

Fig. S7. Cellulase activity assay of PMTSA13^T and OYTSA14. Cellulase activity was assessed on LB agar supplemented with 1% carboxymethylcellulose (CMC) for 3 days, followed by staining with 0.1% Congo red. The presence of a transparent halo surrounding the culture indicates the presence of cellulase activity.

Table S1. Enzymatic activity analysis of PMTSA13^T and OYTSA14. The experiment was conducted by incubating strains in specific enzymatic activity media for the respective enzymes. Amylase activity was assessed on LB agar with 1% soluble starch. Gelatinase activity was determined on LB agar with 1% gelatin. Cellulase activity was evaluated on LB agar supplemented with 1% carboxymethylcellulose (CMC). Catalase activity was assessed on LB agar. Phosphate solubilization activity was determined using Pikovskaya's medium (PVK). IAA (Indole-3-acetic acid) production was detected by incubating strains in Luria-Bertani broth (LB). Nitrogen fixation ability was tested by applying 10 μl drops of liquid culture onto Jensen's medium. The presence (+) or absence (-) of enzymatic activities for PMTSA13^T and OYTSA14 is indicated accordingly.

Activities	PMTSA13^T	OYTSA14
Amylase	-	-
Gelatinase	-	-
Cellulase	+	+
Catalase	+	+
P-solubilization	-	-
Protease	-	-
Siderophore production	-	-
Nitrogen fixation	-	-
IAA production	+	+
